# Knowledge-Fusion-Based Iterative Graph Structure Learning Framework for Implicit Sentiment Identification

**DOI:** 10.3390/s23146257

**Published:** 2023-07-09

**Authors:** Yuxia Zhao, Mahpirat Mamat, Alimjan Aysa, Kurban Ubul

**Affiliations:** 1 School of Information Science and Engineering, Xinjiang University, Ürümqi 830046, China; zyx@stu.xju.edu.cn (Y.Z.); 107552103632@stu.xju.edu.cn (M.M.); alim@xju.edu.cn (A.A.); 2 School of Mathematics and Computer Applications, Shangluo University, Shangluo 726000, China; 3 Engineering Research Center of Qinling Health Welfare Big Data, Universities of Shaanxi Province, Shangluo 726000, China; 4 Xinjiang Laboratory of Multi-Language Information Technology, Ürümqi 830046, China

**Keywords:** knowledge fusion, implicit sentiment, sentiment analysis, graph neural network

## Abstract

Implicit sentiment identification is a significant classical task in text analysis. Graph neural networks (GNNs) have recently been successful in implicit sentiment identification, but the current approaches still suffer from two problems. On the one hand, there is a lack of structural information carried by the single-view graph structure of implicit sentiment texts to accurately capture obscure sentiment expressions. On the other hand, the predefined fixed graph structure may contain some noisy edges that cannot represent semantic information using an accurate topology, which can seriously impair the performance of implicit sentiment analysis. To address these problems, we introduce a knowledge-fusion-based iterative graph structure learning framework (KIG). Specifically, for the first problem, KIG constructs graph structures based on three views, namely, co-occurrence statistics, cosine similarity, and syntactic dependency trees through prior knowledge, which provides rich multi-source information for implicit sentiment analysis and facilitates the capture of implicit obscure sentiment expressions. To address the second problem, KIG innovatively iterates the three original graph structures and searches for their implicit graph structures to better fit the data themselves to optimize the downstream implicit sentiment analysis task. We compared our method with the mainstream implicit sentiment identification methods on two publicly available datasets, and ours outperformed both benchmark models. The accuracy, recall, and F1 values of KIG on the Pun of the Day dataset reached 89.2%, 93.7%, and 91.1%, respectively. Extensive experimental results demonstrate the superiority of our proposed method for the implicit sentiment identification task.

## 1. Introduction

Text sentiment analysis refers to the process of analyzing, processing, generalizing, and reasoning about subjective texts with sentiment using natural language processing (NLP) and text mining techniques [1]. Liu et al. classified sentiment expressions into explicit expressions, which are subjective statements giving sentiment tendencies, and implicit expressions, in which sentiment is implied through objective statements, according to whether the expressions contain obvious sentiment words [2]. Therefore, the implicit sentiment sentence can be defined as “a fragment of language that expresses subjective sentiments but does not contain explicit sentiment words”. Some studies have shown that implicit sentiment expressions account for about 30% of sentiment expressions and are an important part of sentiment expressions [3]. Therefore, the analysis of implicit sentiments is an essential part of sentiment analysis, and an in-depth study of implicit sentiment tasks will help improve the performance of sentiment analysis.

**Figure 1:**
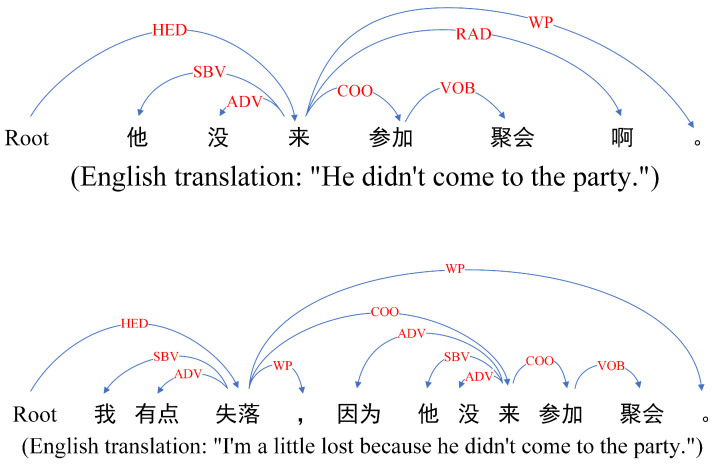
Explicit and implicit sentiment expressions.

As shown in Figure 1, because implicit sentiment is embedded in the semantics of the text, it is usually implicit and unintuitive, and in many cases, the actual sentiment is the opposite of the literal sentiment. This makes the analysis and feature extraction of implicit sentiment difficult, so implicit sentiment analysis has become one of the urgent difficulties in NLP [4].

Recently, graph neural networks (GNNs) have received more attention and attained cutting-edge performance in a variety of NLP tasks, including sentiment analysis [5,6], reading comprehension [7,8], and machine translation [9,10], due to their powerful feature capture capabilities for graph-structured data. When applied to sentiment analysis, GNNs allow direct feature interactions (via relationships between nodes) between different nodes at any location. Specifically, GNN-based approaches first introduce relationships between tokens or sentences explicitly in the graph construction phase (i.e., adding edges). Then, some kind of message passing is used to obtain each node representation by learning information about its neighbors on the aggregated topology [11,12]. Thus, GNN is a promising solution to the problem of implicit sentiment analysis. However, the current approach still suffers from the following two drawbacks:

- **Defects are caused by a single-view graph structure.** Implicit sentiment mining is a non-trivial task, and a suitable text graph structure can accurately represent the structural information among tokens. However, the current graph structure of implicit sentiment text is single and carries a lack of structural information to accurately capture obscure sentiment expressions.
- **Deficiencies arise from predefined fixed graph structures.** The graph structures of GNNs used for text analysis are expected to be plausible enough, but current graph structures are usually extracted from human a priori knowledge, such as syntactic dependency trees, co-occurrence information, etc., which inevitably contain uncertain, redundant, incorrect, and missing edges [13,14]. Since implicit sentiment has no explicit sentiment words, the mining of its sentiment information requires a more accurate topology to represent the semantic information, and this noisy information can seriously impair the performance of implicit sentiment analysis.

To address these issues, in this paper, we introduce a knowledge-fusion-based iterative graph structure learning framework (KIG) for implicit sentiment identification. KIG improves implicit sentiment analysis by learning higher-quality graph structures and node representations through iterative approaches and by using fusion to obtain node representations that synthesize different views. Specifically, for the first problem, KIG constructs graph structures based on co-occurrence statistics, cosine similarity, and syntactic dependency trees through a priori knowledge, providing statistical-based, distance-based, and syntactic-related knowledge, respectively. This provides rich multi-source information for implicit sentiment analysis, which is conducive to capturing implicit obscure sentiment expressions. To address the second problem, KIG innovatively fine-tunes the three original graph structures, aiming to determine their implicit graph structures to make them more relevant to the data themselves to optimize the downstream implicit sentiment analysis task. As the graph structure becomes closer to the graph that is optimal for the implicit sentiment identification task, the iterative approach adjusts when to halt in each small batch.

In summary, our main contributions are summarized as follows:

1. We propose a new implicit sentiment analysis framework (KIG) for the joint iterative learning of graph structures and multi-view knowledge fusion. KIG improves implicit sentiment analysis by fusing multi-view graph structures to obtain an integrated understanding of the knowledge provided by different graph structures.
2. Higher-quality graph structures and node representations are obtained through iterative learning, and when the learned graph becomes close to the optimized graph (for implicit sentiment analysis), KIG dynamically terminates. To the best of our knowledge, KIG is the first attempt to apply an iterative method to implicit sentiment analysis.
3. Our extensive experiments on the benchmark dataset of implicit sentiment and extensive experimental results validate the superiority of our framework.

## 2. Related Work

### 2.1. Implicit Sentiment Analysis

Deep neural network-based approaches have stronger knowledge representation [15]. In recent years, deep neural network-based models, including those based on recurrent neural networks (RNNs) and convolutional neural networks (CNNs), have been widely used in sentiment analysis tasks [16,17,18]. When using RNN models, attention mechanisms are usually introduced to deal with each word in a sentence since it contributes differently to the analysis task [19,20,21]. CNN models, on the other hand, use character-level CNNs to analyze semantic information from the text [22,23]. However, none of these models can effectively capture the dependency tree structure information of sentences. Recently, some research works have analyzed semantic information from the tree structure of sentences [18,24,25], for example, using LSTM or BiLSTM dependency trees or syntax trees for sentiment analysis. Although Tree-LSTM models are able to analyze semantic information from text more accurately, they have difficulty performing parallel computation and have long training times. Recently, Sun et al. [26] proposed a BERT-based fine-tuning model to solve question-and-answer tasks. Wu et al. [27] proposed a context-guided BERT-based fine-tuning approach that uses context-aware self-attentive networks to allocate attention in different contexts. However, these methods do not perform well in implicit sentiment analysis. Some recent research works have started to introduce CNNs into the process of encoding tree-structured information [3,28] to improve model efficiency. In their work, phrase structure trees and grammar-dependent trees were used to encode the semantic information of the target sentence and the context, respectively. Later, Refs. [29,30] used heterogeneous graph convolutional neural networks to mine the information transfer of sentences. As a comparison, previous works used pre-fixed topologies, which may contain some noisy edges. To solve this problem, KIG learns to obtain higher-quality graph structures and node representations by iterative means.

### 2.2. Graph Neural Network Applications

Recently, GNNs have received increasing attention [31]. GNN is a method for learning graph-structured data [32,33]. Currently, researchers studying GNNs usually classify them into two categories: spectral and spatial approaches. The spectral approach uses spectral graph filters [34] to perform convolution operations on the graph domain data, and there are several different filters, such as Chebyshev polynomial filters [32,35]. The spatial approach performs convolutional operations by edge propagation and the aggregation of local information [36,37,38]. This approach not only preserves more topological structure information but also better copes with data in non-Euclidean spaces. Different aggregation functions in spatial GNNs are designed to learn node representations, including LSTM [36], self-attention [37], and summation [38]. These aggregation functions can be adapted and optimized for different task requirements and data characteristics.

In a recent study on related NLP tasks, Yao et al. [11] proposed the construction of a graph structure from a corpus for the classification task. Some researchers converted the nonlinearity between GNN layers into a linear transformation to decrease the complexity of GNNs [39]. Chen et al. [40] added syntactic dependency trees to GNN to represent syntactic structures for sentiment analysis. In earlier research, when constructing graphs, the edges of the graph structure were either based on similarity measures [41], co-occurrence [14], or syntactic structure [13], considering only the information carried by a single graph structure. As a comparison, we consider intra-sentence dependencies, statistical information, and word-to-word similarity measures when constructing the initial graph structures. This provides rich multi-source information for implicit sentiment analysis, thus facilitating the more accurate capture of obscure expressions of implicit sentiment.

## 3. Materials and Methods

### 3.1. Problem Statement

The aim of our work is to learn and mine the implicit sentiment of chapter-level texts. A formal description is as follows:

(1) $${\hat{y}}_{i}=\Theta \left(G_{i}^{1},G_{i}^{2},G_{i}^{3}\right)\in \left\{0,1\right\}$$

Given a set of chapter-level implicit sentiment texts of *N*, multiple-view graph structures $G_{i}^{k}=\left(V_{i},E_{i}^{k},X_{i}\right)$ are first constructed for each text, where *i* denotes the chapter number, *k* denotes the view of the graph structure, $V_{i}$ is the set of nodes of $G_{i}$$\left(\left|V_{i}\right|=n\right)$, $E_{i}^{k}$ is the set of edges of $G_{i}$, $X_{i}\in R^{n\times d}$ is the word embedding matrix of $G_{i}$, and *d* denotes the embedding dimension. Our goal is to output the implicit sentiment identification result ${\hat{y}}_{i}\in \left\{0,1\right\}$ (implicit sentiment, non-implicit sentiment) for each chapter.

### 3.2. Methodology

**Figure 2:**
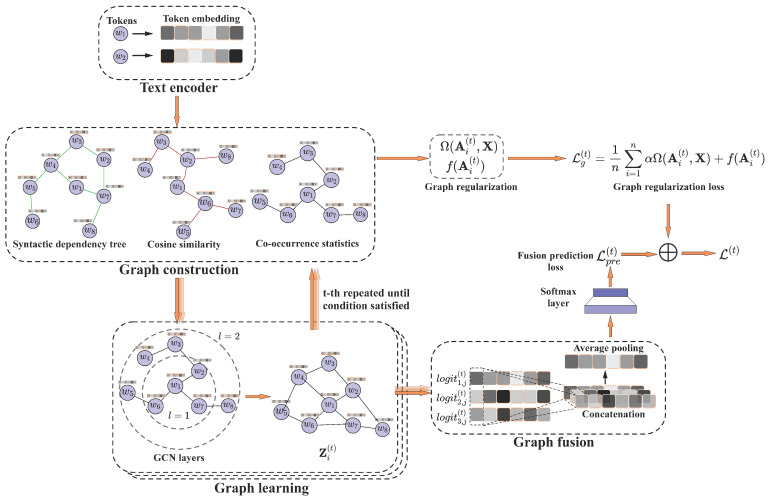
An overview of the proposed KIG framework. KIG consists of four components, i.e., text encoder, graph construction, graph learning, and graph fusion. In the figure, $w_{i}$ denotes token *i*, *l* represents the number of GCN layers, and $Z_{i}^{\left(t\right)}$ represents the representation vector of the hidden space of the graph structure of the *i*-th viewpoint after *t* iterations of optimization. ${logits}_{1,j}^{\left(t\right)}$ denotes the logit of token *j* under viewpoint 1 after *t* iterations.

In this section, we describe the general framework of our proposed KIG, as shown in Figure 2. At the highest level, KIG consists of four components, namely, the text encoder, graph construction, graph learning, and graph fusion. First, the text encoder step maps each token in the implicit sentiment text to a high-dimensional space through a dictionary to obtain a word embedding matrix. After that, the graph construction step constructs original graph structures with three different views to provide multi-source information for the downstream feature extractor. Moreover, the graph learning step performs feature extraction and learns the graph structure in an iterative manner, and the learned node embeddings can provide useful information for learning a better graph structure, aiming to find a suitable graph structure to enhance the original graph structure for the implicit sentiment analysis task. The final step, graph fusion, integrates the features from each view and finds a consistent decision boundary.

#### 3.2.1. Text Encoder and Graph Construction

In the steps described in this subsection, each token in each text chapter is coded with $D=\left\{w_{1},w_{2},...,w_{n}\right\}$, where $w_{i}$ is the token to be encoded. We obtain the word embedding matrix $X\in R^{n\times d}$ by mapping the tokens in the chapter to a high-dimensional vector space, where *n* is the padding length, and *d* is the word vector dimension. Then, we construct text graphs with three different views based on the following knowledge.

**a.**  **Co-occurrence statistics**

Word co-occurrence analysis is widely used in the study of text mining. Positive point-wise mutual information (PPMI) [42] is the re-weighted form of the co-occurrence metric, which is considered to be the state-of-the-art model for measuring the similarity between two words. It can be formulated as follows:

(2) PPMI=max(PMI,0)

(3) $$PMI=\log\frac{\hat{p}\left(w_{i},w_{j}\right)}{\hat{p}\left(w_{i}\right)\hat{p}\left(w_{j}\right)}=\log\left(\frac{\#\left(w_{i},w_{j}\right)}{\#\left(w_{i}\right)\#\left(w_{j}\right)}\cdot \left|E\right|\right)$$

Let $V_{w}$ denote the set of all dataset tokens, $w_{i}\;,w_{j}\in V_{w}$. $w_{i}$ is a central word, $w_{j}$ is a word within the predefined and fix-sized context window L, E is the set of co-occurring word pairs, $\#\left(w_{i},w_{j}\right)$ is the number of times word pair $\left(w_{i},w_{j}\right)$ appears within $V_{w}$, and $\#\left(w_{i}\right)$ and $\#\left(w_{j}\right)$ are the number of occurrences of words $w_{i}$ and $w_{j}$, respectively. So, we can set $A_{ij}=PPMI\left(w_{i},w_{j}\right)$. The relationship between $w_{i}$ and $w_{j}$ becomes closer as the value of PPMI increases.

**b.**  **Cosine similarity**

Implicit sentiment mining is a non-trivial task, and an ideal text representation can accurately capture the implicit linguistic rules and common-sense knowledge hiding in text data, such as lexical meanings and syntactic structures [43,44]. In this component, we use cosine similarity as a metric, because it can measure semantic similarity in terms of lexical meaning and has a wide range of applications. Specifically, the similarity of a word pair is defined by:

(4) $$cos\left(x_{i},x_{j}\right)=\frac{<x_{i},x_{j}>}{\left|x_{i}\left|\cdot \right|x_{j}\right|}$$

where $x_{i}$ and $x_{j}$ are the word embeddings of $w_{i}$ and $w_{j}$, respectively. So, we can set $A_{ij}=cos\left(x_{i},x_{j}\right)$. Similarly to PPMI, the relationship between $w_{i}$ and $w_{j}$ becomes closer as the value of $cos\left(x_{i},x_{j}\right)$ increases.

**c.**  **Syntactic structures**

In this component, we use the syntactic dependency tree as a metric, because it can provide syntactic relations between words. Inspired by [29], we also use the dependency structure as a matrix for the syntactic structure view in this paper. The formal definition is as follows:

(5) $$A_{ij}=\left\{\begin{array}{c} DT\left(w_{i},w_{j}\right)\\ 0 \end{array}\right.\;\;\;\;\;\begin{array}{c} w_{i},w_{j}\;are\;tokens\\ otherwise \end{array}$$

where $DT\left(w_{i},w_{j}\right)$ is the relation between tokens $w_{i}$ and $w_{j}$ in the syntactic structure. When there is a dependency between tokens, $w_{i}$ and $w_{j}$ have connected edges between them. The larger the value of $DT\left(w_{i},w_{j}\right)$, the greater the weight of the edges between the tokens.

#### 3.2.2. Graph Learning

Although the original graph has an unreliable topology, it usually contains lots of useful information. Ideally, the learned graph structure can be used as a complement to the original graph structure. Therefore, we can assume that the learned graph structure is a “fine-tuning” of the original graph structure, and the graph is updated by assembling the learned graph with the original graph.

(6) $${\tilde{A}}_{i}^{\left(t\right)}=\lambda L_{i}^{\left(0\right)}+\left(1-\lambda \right)\left\{\eta f\left(A_{i}^{\left(t\right)}\right)+\left(1-\eta \right)f\left(A_{i}^{\left(1\right)}\right)\right\}$$

As shown in Equation (6), $L_{i}^{\left(0\right)}=D_{i}^{{\left(0\right)}^{-1/2}}A_{i}^{\left(0\right)}D_{i}^{{\left(0\right)}^{-1/2}}$ denotes the adjacency matrix after the original graph normalization of the *i*-th view. $D_{i}^{\left(0\right)}$ denotes the degree matrix of the original state, and $A_{i}^{\left(t\right)}$ and $A_{i}^{\left(0\right)}$ denote the two adjacency matrices at the *t*-th iteration and in the original state, respectively. It is worth mentioning that $A_{i}^{\left(1\right)}$ is calculated based on the original node feature matrix *X*, where $A_{i}^{\left(t\right)}$ is calculated based on the previously updated node embedding $Z_{i}^{\left(t-1\right)}$. We treat the last learned graph structure as a linear arrangement of them, weighted by the balancing parameter λ to combine the advantages of both. We use GCN to map the original node features to the hidden layer space.

(7) $$Z_{i}^{\left(t\right)}=ReLU\left(GCN\left(X,{\tilde{A}}_{i}^{\left(t-1\right)}\right)W_{i}\right)$$

where $Z_{i}^{\left(t\right)}$ is the representation vector of the *t*-th iteration under the *i*-th view of the implicit sentiment chapter, and $w_{i}$ denotes the weight matrix of the *i*-th view. The activation function in this paper is the ReLU function.

#### 3.2.3. Graph Fusion

We aggregate the advantages of the three views by designing a graph fusion module to obtain a consistent document representation. The formalization is shown in Equations (8) and (9):

(8) $$logits=Concat{\left({logits}_{1}^{\left(t\right)},{logits}_{2}^{\left(t\right)},{logits}_{3}^{\left(t\right)}\right)}_{k=1}^{k=d}.$$

(9) $$\tilde{logits}=\frac{1}{3}\sum_{i=1}^{3}\;{logits}_{i}$$

where k∈[1,d] denotes the different dimensional representations of each document, logits denotes the document representation after graph fusion, and the function Concat(·) denotes the operation of stitching by column, whose purpose is to fuse three different views into a consistent representation. After obtaining logits, we finalize the representation of each document by taking the average pooled value, i.e., $\tilde{logits}$.

(10) $$\hat{y}=\sigma \left(\tilde{logits}\right)$$

Next, we decode the representation of each document by using a σ(·) function (Softmax layer), as shown in Equation (10).

Finally, we obtain the graph fusion loss, which is used to reflect the difference between the true value *y* and the graph fusion prediction $\hat{y}$.

(11) $$L_{pre}=-\sum_{n}\;ylog\hat{y}$$

#### 3.2.4. Graph Regularization

Although we are able to improve the quality of document representation by combining different information views, the quality of the learning graph $G_{l}$ usually needs to be considered as well. In practical terms, improving the quality of $G_{l}$ usually requires controlling the connectivity, sparsity, and smoothness of the learning graph, which accurately represents the graph topology between the original word embedding *X* and the implicit sentiment identification task. Consider each column of the word embedding *X* as a graph signal. We assume that the value of the graph signal varies smoothly between adjacent nodes. In this paper, the graph $G_{i}^{\left(t\right)}=\left(A_{i}^{\left(t\right)},X\right)$ after iteration for each view of the document is defined as follows:

(12) $$\Omega \left(A_{i}^{\left(t\right)},X\right)=\frac{1}{2n^{2}}\sum_{r,e}\;A_{i,re}{\|x_{r}-x_{e}\|}^{2}$$

(13) $$f\left(A_{i}^{\left(t\right)}\right)=\frac{-\beta }{n}1^{T}log\left(A_{i}^{\left(t\right)}1\right)+\frac{\gamma }{n^{2}}{\left\|A_{i}^{\left(t\right)}\right\|}_{F}^{2}$$

From Equation (12), it can be seen that minimizing $\Omega \left(A_{i}^{\left(t\right)},X\right)$ enhances the similarity of neighboring nodes, and thus, the graph signal on the adjacency matrix $A_{i}^{\left(t\right)}$ becomes smooth. In addition, the learning graph needs to be added with additional constraints. In Equation (13), ${\left\|\cdot \right\|}_{F}^{2}$ represents the Frobenius norm. The first and second terms are used to control the graph formation and sparsity, respectively. Therefore, the entire graph regularization loss is defined as the sum of the above losses $L_{G}=\alpha \Omega \left(A_{i}^{\left(t\right)},X\right)+f\left(A_{i}^{\left(t\right)}\right)$, where α, β, and γ are hyperparameters.

(14) $$L=L_{pre}+L_{G}=-\sum_{n}\;ylog\hat{y}+\alpha \Omega \left(A_{i}^{\left(t\right)},X\right)+f\left(A_{i}^{\left(t\right)}\right)$$

The final loss function in this paper is shown in Equation (14), enabling it to fuse multi-view information while controlling the connectivity, sparsity, and smoothness of the learning graph.

## 4. Experiments and Analysis of Results

### 4.1. Experimental Setup

#### 4.1.1. Datasets

Pun of the Day (Puns) [45]: Implicit sentiment text was obtained from the website of the same name, and non-implicit sentiment text was obtained from AP News, New York Times, Yahoo News, and Proverbs. The puns contained 2424 implicit sentiment sentences and 2403 non-implicit sentiment sentences, with an average sentence length of 13.5 words.

Reddit [46]: The dataset text was collected using Reddit’s public API. The text is divided into Body and Punchline parts, so the dataset is divided into three types: the Body part dataset, the Punchline part dataset, and the complete dataset, combining Body and Punchline. The dataset contains 13,884 non-implicit sentiment sentences and 2025 implicit sentiment sentences.

We allocated 60% of the Puns dataset to a training set, 20% to a validation set, and 20% to a test set. The Reddit dataset has a total of 15,909 texts, including 14,693 texts in the training set, 608 texts in the validation set, and 608 texts in the test set, as shown in Table 1.

#### 4.1.2. Evaluation Index

To facilitate a comparison with the baseline method, we used accuracy, recall, and F1-score as evaluation indices. We determined the corresponding evaluation index by constructing a confusion matrix, which is shown in Table 2.

In the confusion matrix, TP means that the classifier correctly classifies positive samples as positive cases; FP means that the classifier incorrectly classifies negative samples as positive cases; TN means that the classifier correctly classifies negative samples as negative cases; and FN means that the classifier incorrectly classifies positive samples as negative cases. The specific definitions of accuracy, recall, and F1-score are shown in Equations (15)–(17):

(15) $$Accuracy=\frac{TP+TN}{TP+FP+TN+FN}$$

(16) $$Recall=\frac{TP}{TP+FN}$$

(17) $$F1-score=\frac{2TP}{2TP+FP+FN}$$

### 4.2. Baseline and Parameter Settings

SVM [45]: Support Vector Machine (SVM) is a classical classification algorithm that can be used for implicit sentiment recognition tasks.

HCW2V [45]: HCW2V stands for Hierarchical Convolutional Word2Vec, which is a neural network model for learning word embeddings. The model is hierarchical in nature, meaning that it captures both the local and global contexts of words in a sentence.

TM [47]: Tensor decomposition is used for implicit sentiment recognition.

CNN [48]: A convolutional network is a mainstream neural network, and this author designed a CNN and used it in an implicit sentiment recognition task.

Bi-LSTM+CNN [49]: The Bi-LSTM+CNN model combines two components, LSTM and CNN, which can exploit both long-term dependencies and text features for text classification tasks.

Bi-GRU [50]: GRU is a simplification of LSTM, which is more efficient. In text classification, Bi-GRU can also exploit the long-term dependency and contextual information of the text.

PACGA [50]: The PACGA model can represent speech information and semantic information well for implicit sentiment recognition.

Human (General) [46]: This model, which is comparable to general human performance, is from an Amazon Mechanical Turk study and classifies text by manual scoring.

IDGL [51]: IDGL is a graph neural network framework capable of optimizing the graph structure by deep iteration.

TextCNN [52]: TextCNN is a convolutional neural network model that uses multiple convolutional kernels of different sizes to convolve the input to capture linguistic features of different lengths.

RCNN [53]: A recursive convolutional neural network for text classification applies a recursive structure that captures contextual information while learning word representations.

DPCNN [54]: DPCNN is a model that uses a dilated convolution operation in the convolution layer, thus improving the perceptual field and feature extraction capability of the model.

HAN [19]: HAN is a hierarchical attention network model, mainly used in text classification and sentiment analysis tasks. Multiple attention mechanisms can be built based on the hierarchical structure of the text, resulting in more targeted feature extraction.

In this study, we trained KIG with default parameters. For the word embedding representation, we used GloVe for pre-training, with a word vector of dimension 300, a dropout of 0.5, and a learning rate of 0.001; the optimization method used in this study was adam. To prevent overfitting, we used a learning rate decay and an early stop mechanism during the training process.

In the baseline model of this paper, the results of SVM and HCW2V are cited from [45], the results of TM are cited from [47], the results of CNN are cited from [48], the results of Bi-LSTM+CNN, Bi-GRU, and PACGA are cited from [50], and the results of Human (General) are cited from [46]. No specific parameter settings are given in the original texts for these models, the default parameter settings in the original texts were used, and the parameter settings for all models are shown in Table 3.

### 4.3. Main Results

The experimental results (%) on the Puns dataset are shown in Table 4, where the bolded results indicate the best values, and the underlined ones indicate the second-best values.

The results for the Puns dataset are shown in Table 4, which shows that our model obtained the best results for all three evaluation metrics. In addition, KIG obtained a recall value of 93.72%, which is almost 1% more accurate than the previous best model, the PACCG model. Although the PACCG model can represent the semantic information in the text well, we see that knowledge fusion achieves greater success in extracting key features with more weight by considering multiple sources of knowledge in an integrated manner.

Table 5 shows the results on the Reddit dataset (%), where the bolded results indicate the best values, and the underlined ones indicate the second-best values.

In Table 5, we can see the results of the experiments conducted using the Reddit dataset. We ran our model on the Body part, the Punchline part, and the Full text, respectively. On the Full dataset, we find that KIG can achieve an accuracy of 67.84%, while TEXTCNN achieves 67.10%. We also note that the average human identifies implicit sentiment text with an accuracy of around 66.30%.

To learn more about what the model does with the dataset, we used only the Body and Punchline datasets to see which part of the text is more useful for implicit sentiment identification. We found that most of the deep learning methods rely more on the Body part of the text in their predictions, while ordinary humans rely more on the Punchline part. This result may be due to the fact that deep learning models can extract more features from the Body part compared to the Punchline part, while humans are more likely to identify sentiment through the Punchline part.

### 4.4. Ablation Experiment

To further evaluate the degree of impact of each module of the model in this paper on its performance, we further investigated by performing ablation experiments. First, we conducted experiments on the single-view graph structure and the multi-view fused graph structure to evaluate the contribution of the fused graph structure to KIG. Moreover, we also removed the iterative optimization module and the graph regularization module separately to evaluate the impact of these two modules. The specific experimental results are shown in Table 6.

We performed an ablation study to evaluate the impact of different model components. In Table 6, w/o IL denotes without iterative learning, and graph reg. denotes graph regularization. We conducted ablation experiments for the model on the Puns dataset and the Reddit dataset. By comparing the experimental results of the single-view graph structure with the multi-view fusion graph structure of the whole model, we can see that the multi-view fusion graph structure improves the performance of the model, which proves that the proposed knowledge fusion framework is helpful for the original graph construction. By turning off the iterative learning component, we can see that the performance of the model significantly decreases, which proves the effectiveness of the proposed iterative learning framework for graph learning problems. We can also see the benefit of using graph regularization loss to jointly train the model.

### 4.5. Parameter Analysis

The graph regularization step usually requires controlling the smoothness, connectivity, and sparsity of the resulting learning graph to serve the purpose of improving the quality of document representation. To explore the effect of the hyperparameter smoothness_ratio α and sparsity_ratio γ of this model on the model results, we performed a sensitivity analysis on the model accuracy by controlling the ranges of α and γ using a grid search.

**Figure 3:**
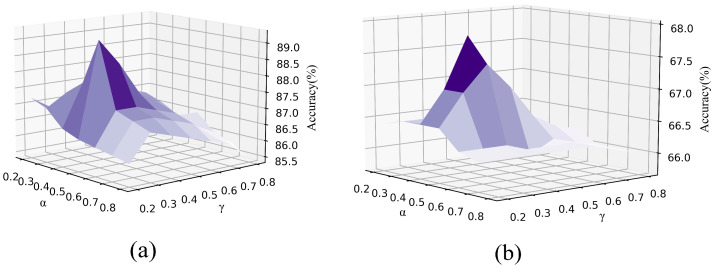
Visualization of parameter sensitivity analysis. (**a**) Puns dataset and (**b**) Reddit-Full dataset.

We conducted experiments on the Puns dataset and the Reddit-Full dataset. The results indicating the variation in model accuracy with α and γ are shown in Figure 3. From Figure 3, we find that the visualization plots of the two datasets are very similar. With the increase in the smoothness_ratio α, the accuracy of the model first increases and then decreases. Therefore, controlling the smoothness of the adjacency matrix can improve the quality of document representation, but excessive smoothing will lead to the excessive sparsity of the adjacency matrix, which in turn affects the accuracy of the model. We can also see from the variation in the accuracy value with γ that the accuracy decreases substantially as the sparsity_ratio γ increases. This is because an excessively sparse graph structure will lose the semantic information of the text. Therefore, the graph regularization phase plays an important role in the implicit sentiment identification task of the model.

### 4.6. Number of Iterations

In the method described in this paper, the iterative process plays an important role when the model is in the graph learning step. We can search for an implicit graph structure to enhance the original graph structure for the implicit sentiment analysis task. To demonstrate the role of the iteration module on the model in this paper, we visualized the effect of the number of iterations on the accuracy of the model on the Puns dataset.

**Figure 4:**
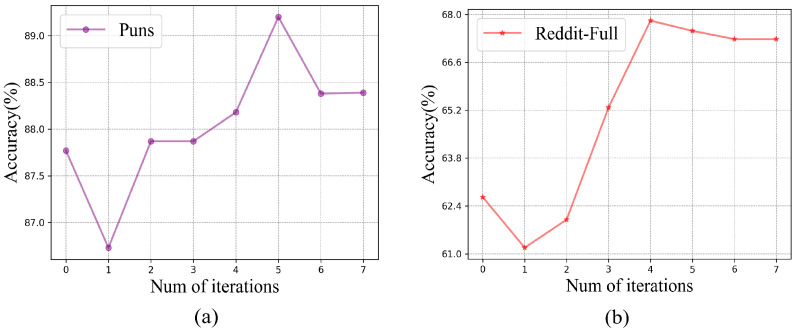
Convergence of model iterations. (**a**) Puns dataset and (**b**) Reddit-Full dataset.

We validated the effect of the number of iterations on the accuracy of the model on the Puns dataset and the Reddit-Full dataset. From Figure 4, we can see that the accuracy of the model decreases when it is first added to the iterations, but it gradually increases and finally reaches convergence as the number of iterations increases. This result may be due to the fact that the model will be unstable when it is first added to the iterations, but as the number of iterations increases, the model will tend to converge. Since we only use GCN as the underlying GNN module of KIG in our experiments, this convergence is not caused by the smoothing property of GNN. Our deep iterative process of graph structure learning allows the learned adjacency matrix to be greatly optimized for the implicit sentiment analysis task. This verifies that iterative learning plays a significant role in the model in this paper.

In addition, the accuracy of the model in Figure 4 decreases slightly after reaching the maximum value in all cases, which may be due to the overfitting phenomenon of the model, but our model uses a dynamic stopping mechanism that stops iterating when the graph structure suitable for the downstream task is learned iteratively, which can effectively mitigate this situation.

### 4.7. Case Study

The advantage of KIG is that the original graph structure from different views is constructed in the graph construction step for graph fusion to provide multiple sources of information for the downstream feature extractor, and the graph structure is learned iteratively in the graph learning step.

**Figure 5:**
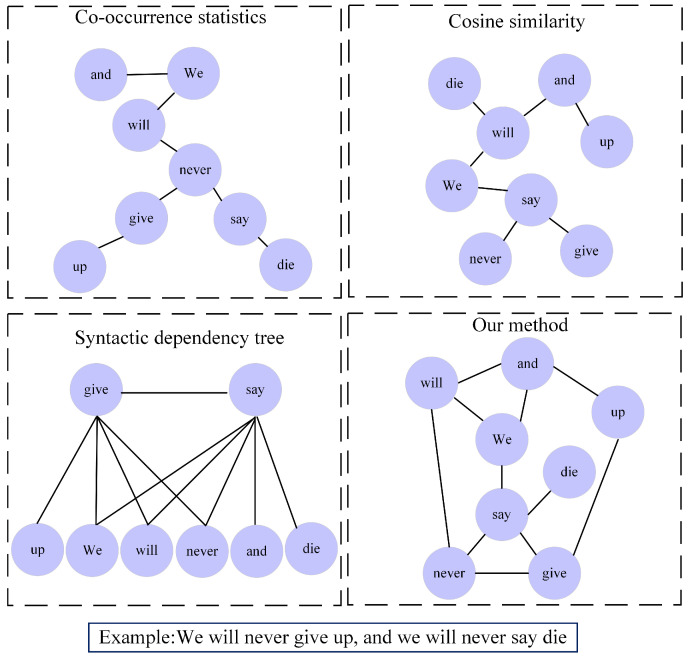
Case study visualization diagram.

In Figure 5, we use a text as an example based on the views of co-occurrence statistics, cosine similarity, and syntactic dependency trees from the co-occurrence statistics of tokens, cosine similarity after tokens are transformed into word vectors, and syntactic relations between tokens, respectively. The single-view graph structure usually constructs a corresponding graph structure based on a single rule for the text, which can only consider the feature information of different texts to a certain extent, and the graph structure is sparse and cannot represent the semantic information well. Our model, however, can take into account various aspects of the text by fusing graph structures from different views. Since the multi-view information of different graph structures is fused, the weights of edges between similar nodes in the graph structure increase, and then the redundant noisy edges are removed through iterative learning to obtain a more stable graph structure, which can better learn node embeddings for downstream implicit sentiment identification tasks. For example, by comparing the text information in Figure 5, we find that our method not only considers the cosine similarity between the tokens of the text and connects the two lexically similar tokens “and” and “up” but also incorporates the structure that is present in both co-occurrence statistics and syntactic dependency trees to connect “give” and “up” and also removes the edges between the two less related tokens “will” and “die” through iteration to obtain a more stable structure for building graphs. Therefore, our method can better represent the semantic structure of the text and provide more effective information for implicit sentiment analysis. This shows that KIG fuses graph structures according to different views, and iterative learning of the original graph structure has an important role in the whole model.

### 4.8. Error Analysis

From the results of the model, we selected a total of 40 misidentified texts for error analysis. Among them, 20 were false negatives (implicit sentiment texts were identified as non-implicit sentiment texts), and 20 were false positives (non-implicit sentiment texts were identified as implicit sentiment texts). At the highest level, KIG consists of four components, namely, the text encoder, graph construction, graph learning, and graph fusion. In the graph construction step, we note that although KIG can provide multi-source information to the downstream feature extractor through three different views of the initial graph structure, each view of the graph structure inevitably generates some noisy edges. For example, in the text “I used to be a banker but I lost interest”, the syntactic dependency tree view connects the edges of “to” and “banker”, while neither the cosine similarity view nor the co-occurrence statistics view connects these two tokens, which may be a noisy edge. In the graph learning step, if these noisy edges are not removed by iterative learning, the graph fusion step will increase the noisy edges of the fused graph by fusing the graph structures of different views, which will have a more serious impact on the downstream implicit sentiment identification task. Therefore, we need to focus more on building better-quality graph topologies that contain a more semantic structure of the text in the graph construction step. The graph learning step also occurs through iterative learning, which makes the text graph structure more stable and robust; this is key to our model, and it is also an effective prerequisite for fusing less noisy information and finding more consistent decision boundaries in the graph fusion step.

## 5. Conclusions and Future Work

In this paper, we propose a knowledge-fusion-based iterative graph structure learning framework (KIG), which can iteratively optimize the original graph structure to obtain an implicit graph structure that is more adaptable to the downstream implicit sentiment analysis task. It can also provide rich multi-source information and increase the expressiveness of implicit sentiment by fusing graph structures from different views. On the Puns dataset, KIG achieved about 0.5%, 0.9%, and 0.3% improvement in accuracy, recall, and F1 values, which increased to 89.2%, 93.7%, and 91.1%, respectively, compared to the baseline model. The experimental results show that the fusion of multi-view graph structures can synthesize the knowledge provided by different graph structures, and the iterative optimization of graph structures can effectively improve the performance of the model for implicit sentiment analysis tasks. In the future, it will be necessary to obtain graph structures that are more consistent with the characteristics of the data from more different views for implicit sentiment text and to fuse different graph structures more effectively. In addition, the need to optimize the original graph structure using labels, which requires a certain cost, and perform unsupervised learning of graph structures without using labels is also a direction we need to study in the future.

## Figures and Tables

**Table 1:** Data statistics for both datasets.

Dataset	Implicit Sentiment	Non-Implicit Sentiment	Average Length	Train	Dev	Test	Total
Puns	2424	2403	13.5	2897	965	965	4827
Reddit	2025	13,884	17	14,693	608	608	15,909

**Table 2:** Confusion matrix.

Actual/Predicted	Positive	Negative
Positive	True Positive (TP)	False Negative (FN)
Negative	False Positive (FP)	True Negative (TN)

**Table 3:** Parameters of all models.

Model	Epoch	Batch_Size	Max_Length	Learning_Rate	Dropout_Rate
DPCNN	5	128	200	0.001	0.2
IDGL	30	16	50	0.001	0.5
HAN	5	128	200	0.001	0.2
TEXTCNN	10	128	200	0.001	0.2
RCNN	10	128	200	0.001	0.2
KIG (ours)	50	16	50	0.001	0.5

**Table 4:** Results of three evaluation metrics for Puns dataset. The best and second-best results are shown in bold and underlined, respectively.

Methods	Accuracy (%)	Recall (%)	F1 (%)
TM	74.50	72.30	73.70
SVM	83.85	82.52	84.18
HCFW2V	85.40	88.80	85.90
Bi-LSTM+CNN	85.38	91.97	86.37
CNN	86.10	86.40	85.70
Bi-GRU	87.72	92.46	88.15
PACGA	88.69	92.76	90.81
KIG (ours)	**89.21**	**93.72**	**91.15**

**Table 5:** Results of Accuracy evaluation metrics for the Reddit dataset. The best and second-best results are shown in bold and underlined, respectively.

Methods	Body (%)	Punchline (%)	Full (%)
Human (General)	49.30	59.20	66.30
DPCNN	56.74	55.42	57.73
IDGL	60.36	57.56	61.67
HAN	61.18	63.48	64.14
TEXTCNN	61.84	66.11	67.10
RCNN	63.98	63.81	64.63
KIG (ours)	**64.37**	**64.25**	**67.84**

**Table 6:** Results of ablation experiment. Best results are in bold.

Dataset	Puns	Reddit-Full
Evaluation index	Acc(%)	Acc(%)
Single graph	86.23	60.19
w/o IL	87.78	62.66
w/o graph reg.	86.12	59.70
Full-model KIG	**89.21**	**67.84**

## Data Availability

Not applicable.
